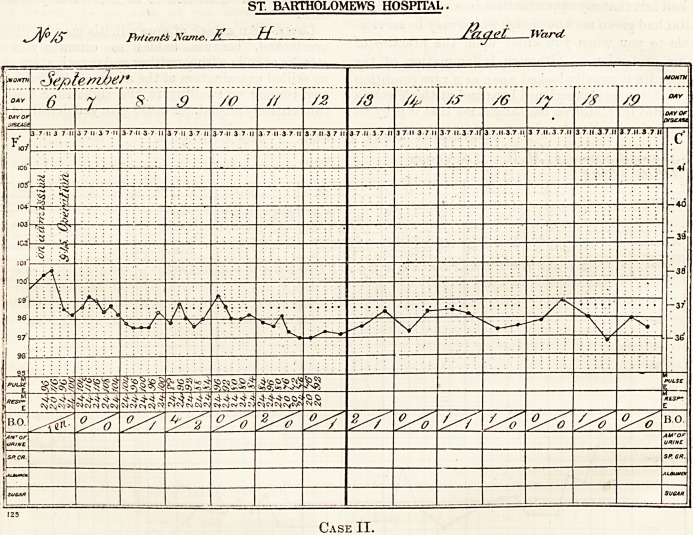# Appendicitis in Children

**Published:** 1905-11-04

**Authors:** D'Arcy Power

**Affiliations:** Surgeon to St. Bartholomew's Hospital and to the Bolingbroke Hospital. Senior Surgeon to the Victoria Hospital for Children, Chelsea


### Hospital Clinics.

### APPENDICITIS IN CHILDREN.

By D'Arcy Power, F.R.C.S.Eng., Surgeon to St. Bartholomew's Hospital and to the Bolingbroke Hospital. Senior Surgeon to the Victoria Hospital for Children, Chelsea.

(A Clinical Lecture delivered at St. Bartholomew's Hospital, November 1, 1905.)

So much has been written about appendicitis that I should not have ventured to take up your time with the subject this afternoon, gentlemen, unless I had felt that my opportunities in a children's hospital had given me knowledge which may be serviceable to you when you enter upon the practice of your profession. Although inflammation of the appendix is usually looked upon as a rare condition in children, my experience here and at the Victoria Hospital for Children has taught me that it is by no means so uncommon as is generally supposed. But it is certainly rarer in London than it is at the present time in Paris, for when I was in that city in May last I made it my business to go round the hospitals for sick children and to ask questions of all my friends who are practising children's surgery there. I learnt that in the hospitals, as well as in private practice, appendicitis in children is extremely common, and that the tendency is for the disease to become more rather than less frequent. This is true also in London, for the number of cases admitted into our hospitals is increasing yearly, though we do not yet see nearly as many as our colleagues in France.

An attack of appendicitis in children does not run quite the same course as in adults; various conditions may mask the diagnosis; the prognosis differs, and the treatment is not identical. I wish to direct your attention to each of these points in turn.

*Cases.*—An attack of appendicitis may be wholly overlooked, because babies so commonly have attacks of colic with vomiting and constipation that no skilled examination of the abdomen is made, and any tension or swelling in the right iliac fossa is thus entirely missed. I could give you details of the case of a boy aged four months who was admitted under my care with symptoms of acute intestinal obstruction, on whom I operated and found that at some time previously the child had suffered from appendicitis, for the appendix was surrounded by adhesions, one band of which, stretching across the ascending colon, was attached to the parietal layer of the peritoneum. This band constricted the ascending colon, and had caused its strangulation. I divided the band, removed the appendix, and the baby made an uninterrupted recovery.

Case I.

No two cases of appendicitis in children are quite alike even in adults, but there is a certain similarity between them which makes the diagnosis fairly easy. In children, on the other hand, this general similarity is less often present. Even the worst cases, too, do not suffer from the same symptoms as adults. It is necessary, therefore, for the practitioner to pay especial attention to such signs as may be present, for if he depends only or chiefly upon the symptoms, he will be misled both in his treatment and in his prognosis. The following cases have impressed this on my mind in a very forcible manner:

Case I.—A girl aged six years was admitted into my wards with a history that ten days previously she had suffered from a cough and had pleurisy. She then complained of pain in her stomach and was sick, but she was better on the following day, though she still had a stomach-ache, which became worse when she passed her water. Her temperature was not raised, and her bowels were open daily. I saw her directly after her admission to the hospital, where she came with a diagnosis of appendicitis made by her medical man. The accompanying chart shows how misleading was her temperature. Two days later her general condition improved greatly. She had not vomited, there was no superficial tenderness, and her bowels had been open daily without enemata, whilst her abdomen was quite soft and was not distended. The only disquieting features were that her pulse never sank below 104, and her respirations varied between 36 and 24 a minute. She remained in this condition for two days longer, when she suddenly became collapsed, her temperature sinking to 96.6° F., her respirations increasing to 44 and her pulse to 132 (see chart). She died, and the post-mortem examination showed a gangrenous appendix with general peritonitis.

Now this was a thoroughly misleading case from the beginning, and the more especially as regards treatment. Our diagnosis that the child had appendicitis was correct, and we ought to have been warned by the continued rapidity of the pulse and respirations to explore the appendix. But the general aspect of the patient, her good physical condition, the freedom with which the bowels acted without medicine, the low temperature, and the absence of more than the slightest physical signs in the right iliac region led me to postpone an operation, which, as the result showed, was urgently needed from the beginning.

Case II.

Case II.—But the warning was not entirely lost upon me, as may be seen by the following case: a girl aged ten years was admitted into Paget Ward on September 6, 1905, with the history that on September 1 she had begun to limp, and when questioned by her mother, she said that she had a pain on the right side of her stomach. The pain continued, and was worse at night, but the patient went to school as usual until September 5. She had not been sick, but her bowels after September 3 were only open when an aperient had been given. The temperature, as in the former case, was misleading, and she did not appear to be in much pain when I saw her, for she lay comfortably in bed, looking bright and with a good colour. I kept her under observation for nine hours, and during that time her pulse-rate rose from 96 to 120, though she was lying quietly in bed. Guided by the rise in the pulse-rate I opened the abdomen. A considerable quantity of pus escaped, which was not very offensive, and a concretion was found lying loose in the abscess-cavity. The appendix was found without much trouble and was removed. A drainage tube was passed to the bottom of the abscess, and was removed on the following day, when it was replaced by a light packing of gauze. The child made an uneventful recovery, and was discharged on October 6.

Now this child did not seem very ill, either to her parents or to the medical man who saw her, yet the appendix had perforated, a concretion had escaped, and there was a good-sized abscess. The condition was accompanied by such slight symptoms that it was only the sad experience of the previous case which led me to recognise the danger and to be very firm in advocating the necessity for an immediate operation.

In two subsequent and similar cases of appendicitis in children I have allowed myself to be guided by the pulse and the respirations rather than by the physical signs, the appearance or the temperature of the patient. The operation in both cases was justified by the result. In the one there was a perforated appendix lying in an abscess, which differed from the ordinary appendix abscess by the absence of smell, though there was plenty of pus; whilst in the other case the appendix was a greatly distended bag of pus, which had not yet burst. There was the same sequence of symptoms in both these children as in the first and fatal case—namely, a quick pulse, rapid respirations, a nearly normal temperature, and very slight local signs in the abdomen. The inodorous character of the pus explains perhaps the absence of more marked symptoms, and I am sorry that the surroundings of the operations—hurriedly, after midnight, and in private houses—prevented any bacteriological or histological examination.

*Signs and Symptoms.*—There are several facts to be learnt from these cases. First, about the manner of onset and the early symptoms and signs. The onset is often gradual, and although the child complains of stomach-ache, the pain is not sufficiently severe to arose much attention or to prevent attendance at school. It may thus be differentiated from the more severe initial symptoms of enteritis and intussusception. Constipation and vomiting, which are characteristic symptoms in the appendicitis of adults, are by no means so common in children; indeed, in the two cases I have quoted the bowels were open naturally throughout the whole illness, and the children were not sick. I do not wish to be understood to say that children with appendicitis are not sick and constipated—as a rule they are—but the exceptions to the rule are more numerous in children than in adults. I think, too, that appendicitis is less frequently a primary affection in children than in adults. There is often a history in children that the patient has been under treatment for broncho-pneumonia, pleurisy, or influenza a week or two before the attack of appendicitis began, whilst in adults an inflammation of the appendix frequently begins without warning. The nervous system of children is much more sensitive than that of adults, and they refer their sensations much more readily than we do. It happens, therefore, as in the second case, that the child is sometimes seen to limp at the beginning of an attack of appendicitis even before there is much complaint of pain, whilst pain on passing water and difficulty of micturition are quite common symptoms. There is a difference also in the decubitus which may easily mislead those who have not devoted much attention to the diseases of children. An adult with appendicitis generally lies flat on his back in bed, with his knees a little bent to relieve the tension of his abdominal muscles; a child as often as not lies curled up on his right side, and with his right thigh more flexed than his left.

As a rule, the local signs of appendicitis are more easy to discover in a child than in an adult, because the abdominal walls are thinner; but the tonic contraction of the muscles may be reduced to a minimum, the local tenderness may be slight, and the rectal examination may reveal nothing even in the worst cases. The pulse and respirations, therefore, are of greater prognostic value than the physical signs. It is not unusual, on the other hand, to have great induration in the region of the appendix—an induration which gradually subsides after a longer or shorter interval without any evidence of suppuration. When suppuration occurs I have often noticed that the abscess is situated higher up in the abdomen and nearer to the umbilicus than the appendix abscess in adults. This is due, in part, to the smaller size of the abdominal cavity in children, but chiefly, I think, to the fact that deviations from the normal position of the appendix are potnet factors in the liability to inflammation. These alterations in position or structure may be acquired, or, as in children, they may be congenital. The children with the most marked deviations from the normal type suffer earliest from appendicitis if the other factors be present. The deviations which are most likely to cause appendicitis are those in which the appendix lies parallel to the posterior wall of the cæcum, where it is often shut off by the adhesion of the folds forming the ileo-colic fossa. An examination of the cæcum in many different individuals shows that it presents two great types. The one, a simple form in which it tapers off into the appendix; the other, a more highly-developed form, where the end is rounded or lobulated and the appendix comes off from it abruptly. When the cæcum is lobulated the appendix often arises from the posterior wall, and it may then be directed outwards, so that an appendix abscess may be extra-peritoneal or inwards when the abscess might be situated in the very middle of the abdominal cavity. When I was preparing my Hunterian lectures on intussusception in 1897 I examined the cæcum in sixty-four children in the post-mortem room at the Victoria Hospital, grouping them, as was done by Treves, into the types A, B, C, and D. The lobulated type of cæcum (or type C) was the least frequent, as it only occurred six times in the sixty-four cases. But the observation shows why it is that an appendix abscess in children is sometimes placed more centrally and higher in the abdomen than is usual.

The skins of children are more delicate and more active than the skins of adults. There is, too, a closer connection between the alimentary canal and the integument in children. The so-called "enema" rash is not uncommon in children with appendicitis, and I have seen it when no enema has been given. It occurs as a rash over the face, chest, and abdomen, which is of the nature of an erythema, and varies from the wheals of true urticaria to a diffuse redness suggestive, at first sight, of measles or scarlatina. The rash appears to be due to intestinal disturbance or to the absorption of inflammatory products, for I have seen it most often during the period of resolution when a large inflammatory mass in the right iliac fossa has begun to diminish in size without suppuration, and after the removal of an appendix in the quiet stage when many adhesions have had to be broken down during the operation The rash is readily distinguished from the other erythemata which it resembles superficially by the fact that its appearance is not attended with any rise of temperature, and it may even be coincident with a fall in the temperature.

(*To be continued.*)

## Figures and Tables

**Case I. f1:**
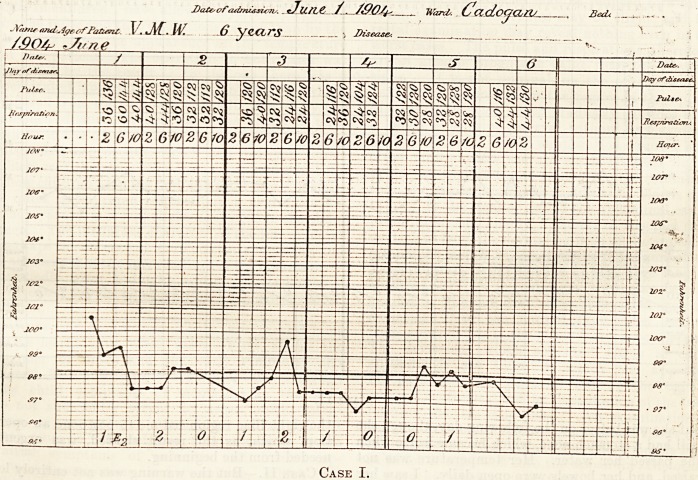


**Case II. f2:**